# Botox-Enhanced Stellate Ganglion Blockade for the Treatment of Post-traumatic Stress Disorder

**DOI:** 10.7759/cureus.37573

**Published:** 2023-04-14

**Authors:** Jonathann Kuo, Megan Nicklay

**Affiliations:** 1 Pain Management, Hudson Health, New York, USA

**Keywords:** ptsd symptoms, somatic symptoms of anxiety disorders, stellate ganglion block (sgb), botulinum (botox), ptsd diagnosis and treatment, post traumatic stress disorder (ptsd)

## Abstract

Emerging evidence promotes stellate ganglion blocks (SGB) as a treatment for post-traumatic stress disorder (PTSD) in individuals who have not fully responded to conventional therapies. Ongoing research aims to assess the reliability and sustainability of this intervention. A 36-year-old female presented to our clinic complaining of severe and persistent symptoms since childhood, consistent with a diagnosis of PTSD and trauma-induced anxiety. The patient tried traditional psychological therapies and psychotropic medications for multiple years without optimal symptom relief. The patient underwent two sets of bilateral SGB: one set of standard injections performed with 0.5% bupivacaine and one set performed with the addition of botulinum toxin (Botox) injected into the stellate ganglion. After the initial standard bilateral SGB procedures, the patient experienced a significant reduction in PTSD symptoms. Two months later, however, the somatic symptoms of PTSD and trauma-induced anxiety returned, including hypervigilance, nightmares, insomnia, hyperhidrosis, and muscle tension. The patient elected to proceed with a set of Botox-enhanced SGB, and the results demonstrated profound relief as quantified by a drop in PTSD Checklist Version 5 (PCL-5) scores from 57 to 2. At a six-month follow-up since the initial injections, the patient reported significant and sustained relief from her PTSD symptoms. We report that the addition of Botox in a selective blockade of the stellate ganglion reduced our patient’s PTSD symptoms to below the PTSD diagnostic threshold for a sustained period while providing additional benefits of reduced anxiety, hyperhidrosis, and pain. We provide a reasonable explanation for our findings.

## Introduction

Post-traumatic stress disorder (PTSD) is a debilitating condition characterized by re-experience and avoidance symptoms that can adversely affect an individual’s quality of life, including intrusive thoughts, nightmares, problems with sleep and concentration, irritability, increased reactivity, increased startle response, and hypervigilance [1]. The signs and symptoms of PTSD reflect a chronic dysregulation of sympathetic reactivity and activity both during mental stress and under resting conditions [2]. Based on the hypothesized mechanism of stellate ganglion blocks (SGB) as a down-regulator of sympathetic nervous system activity, continued research has supported SGB as a therapeutic avenue for individuals with PTSD and trauma-related anxiety [3-4]. Research studies have demonstrated encouraging results for SGB as a treatment for PTSD, yet, in some cases, the limited extent or duration of relief may impact longitudinal outcomes. We proposed that botulinum toxin (Botox) would prolong the inhibition of the transmission of nerve impulses in the sympathetic nervous system, thus prolonging relief from symptoms of PTSD and trauma-related anxiety.

Several case reports and pilot studies have examined the effectiveness of Botox injections as sympathetic blocks to prolong relief from complex regional pain syndrome (CRPS) and hyperhidrosis. Botulinum toxin blocks cholinergic transmission and sympathetic outflow if administered near the sympathetic ganglia and may be used in sympathetically maintained pain control as a sympatholytic [5]. A randomized, blinded study revealed that Botox-enhanced lumbar sympathetic blockades were significantly more effective at reducing visual analog scale pain scores in CRPS patients, providing analgesic effects for an average of two months longer when compared to blockades with local anesthetic alone [6]. Furthermore, a 2021 case report concluded botulinum toxin injection into the stellate ganglion was simple and safe and produced longer-lasting effects than a single nerve block. In this case, a patient with uncontrolled craniofacial hyperhidrosis underwent several standard stellate ganglion blocks, each providing temporary symptom relief. Additionally, the patient received an SGB with pulsed radiofrequency treatment, improving symptoms for more than seven months until sweating returned with greater severity. Finally, Botox was injected into the stellate ganglion, reducing excessive sweating symptoms for over six months with no reported adverse effects [7].

Developing similar modifications to the traditional SGB procedure that extend the length of its clinical benefits would have profound implications for a subset of PTSD patients. We herein report a case in which Botox was injected into the stellate ganglion of a patient with PTSD, providing profound and sustained relief of symptoms.

## Case presentation

A 36-year-old female with a history of PTSD, depression, and anxiety from childhood traumas and abuse presented for initial SGB treatment for PTSD. The patient reported symptoms of insomnia, extreme irritability, hypervigilance, hyperhidrosis, chronic pain, and muscle tension. The patient reported the persistence of these symptoms for multiple years and increased symptom severity with traumatic triggers and stress despite psychiatric and psychological interventions. The patient was currently under the care of a psychiatric nurse practitioner and a therapist. Her practitioners prescribed Celexa 40 mg and Seroquel 50 mg for depression and anxiety. The patient had also tried a myriad of other prescription psychotropics since her teenage years, including selective serotonin reuptake inhibitors, atypicals, and various other medications, which caused adverse reactions of hypersomnia and weight gain. The patient also tried multiple therapeutic modalities such as talk therapy, device-guided breathing exercises, psilocybin, somatic therapy, dialectical behavior therapy, and massage therapy. She also noted a prior history of seizures, the last episode being 18 months before her initial evaluation in our clinic. She stated her previous doctors suspected these to be trauma-related, as scans and testing did not reveal any reason for them to occur.

The patient received one standard bilateral SGB treatment and one bilateral Botox-enhanced SGB treatment over six months at our clinic. During this time, she continued talk therapy and maintained her prescribed medication regimen. While the patient perceived these methods to be minimally helpful, there remained a possibility for confounding or synergistic benefit of these therapeutics when combined with SGB treatment. In addition to completing a detailed medical and personal history questionnaire, she completed the PTSD Checklist Version 5 (PCL-5), a 20-item self-report measure that assesses the severity of 20 PTSD symptoms based on the Diagnostic and Statistical Manual of Mental Disorders, Fifth Edition (DSM-5) [8]. According to guidelines for using the PCL-5, a total score of 31-33 or higher indicates probable PTSD and suggests the patient may benefit from PTSD treatment. The PCL-5 assesses patient symptoms in the past month, so repeated administrations more or less frequently than once a month may be used to track symptom changes and to help determine the appropriate next steps or treatment options. Before the initial treatment, the patient had a PCL-5 score of 57 points, above the clinical threshold of 31 points used to give a PTSD diagnosis. SGB has been well-studied as a safe and effective procedure indicated for PTSD treatment [9].

Treatment series 1: dual sympathetic blocks (bilateral)

The patient presented for initial treatment with primary symptoms of hypervigilance, insomnia, anxiety, and nightmares. Based on these symptoms, an initial PCL-5 score of 57, and a previous PTSD diagnosis, the patient underwent bilateral SGB procedures. The preferred modality here is the dual sympathetic block (DSB) of the stellate ganglion, which is performed at the sixth cervical vertebra (C6) and fourth cervical vertebra (C4) levels (two levels) on both the right and left sides of the cervical spine within one week. The DSB approach has been consistently more clinically effective than a single-level, single-sided SGB [10-11]. According to site protocol, we injected 7 mL of 0.5% bupivacaine into and around the sympathetic ganglion at the right C6 anterior tubercle level and 3 mL of 0.5% bupivacaine at the right C4 anterior tubercle level. The patient tolerated the procedure without complications and was monitored in the postop area for 30 minutes with hemodynamic monitoring. During this time, signs of ipsilateral Horner's syndrome (ptosis, miosis, enophthalmos, facial anhidrosis, and conjunctival injection) developed. The signs of Horner's syndrome were evidence of a successful cervical sympathetic blockade [4].

One week later, the patient ​​presented for follow-up and a repeat DSB on the contralateral side [11]. The patient reported over 75% improvement in symptoms, noting particularly great relief from physiological responses to anxiety and insomnia. The patient reported only one episode of nightmares in the past week when these were occurring nearly every night before the initial right-sided DSB. Her repeat PCL-5 checklist score dropped from 57 to 15 (Table 1). A decrease of 10 points or more is considered a clinically meaningful response to treatment [8].

**Table 1:** PTSD Checklist Version 5 (PCL-5) score changes from the time of treatment to one week A decrease of 10 points or more is considered a clinically meaningful response to post-traumatic stress disorder (PTSD) treatment. While the patient responded meaningfully to both treatment series, we report that the addition of botulinum toxin (Botox) in a selective blockade of the stellate ganglion (SGB) sustained symptom relief for a longer duration.

	PCL-5 Score at Time of Treatment	PCL-5 Score at One Week	Change in PCL-5 Score at One Week
Standard SGB	57	15	-42
Botox-enhanced SGB	14	2	-12

One month later, the patient followed up via telehealth when she continued to report relief from PTSD symptoms, which she attributed to the SGB treatment. Her symptoms of intrusive thoughts resolved, and her overall PCL-5 score remained decreased to eight at this time. Since undergoing the initial treatments, the patient secured a new job and an apartment. Despite the positive impact on her quality of life, the patient continued to experience somatic symptoms of anxiety, insomnia, and hyperhidrosis.

Treatment series 2: Botox-enhanced stellate ganglion blocks (bilateral)

Two months after the initial treatment series, the patient returned to our clinic concerned about recurrent symptoms after a limited duration of relief. Despite having clinically significant initial results from the standard SGB treatment, there was a notable 75% increase within two weeks, as her PCL-5 score began increasing back up to 14, reflecting recurrent PTSD symptoms of hyperreactivity, insomnia, sweating, and muscle tension (Table 2). Given the patient’s complex history of trauma and treatment-resistant psychological symptoms, we considered alternative methods to address these recurrent symptoms that the patient considered severe and impairing. Although the PCL-5 score at this time remained below the clinical threshold for diagnosing PTSD, the current guidelines state a 5-10 point change reflects a reliable shift in existing or new symptoms for which additional treatments may be indicated [12]. The patient wished to extend the duration of the positive effects she felt from the standard SGB treatment. Based on the evidence discussed above, Botox may prolong the therapeutic outcomes of a sympathetic block beyond the length of relief provided by an anesthetic alone [6]. After consultation and discussion of the risks and benefits, she elected to undergo a repeat right SGB with the additional injectant of 50 units of Botox into the stellate ganglion and to receive a second 50-unit Botox-enhanced SGB on the left side two weeks later.

**Table 2:** PTSD Checklist Version 5 (PCL-5) score changes from the time of treatment to two months A 5-10 point change in PCL-5 score indicates a reliable shift in existing or new symptoms. The six-point increase between six weeks to two months following the standard stellate ganglion blocks (SGB) reflected the patient’s concerns about recurrent post-traumatic stress disorder (PTSD) symptoms. The patient elected to repeat the SGB procedures with the addition of botulinum toxin (Botox), hoping to extend the duration of her initial symptom relief.

	PCL-5 Score at Time of Treatment	PCL-5 Score at Six Weeks	PCL-5 Score at Two Months	Change in PCL-5 Score from Six Weeks to Two Months
Standard SGB	57	8	14	+6
Botox-enhanced SGB	14	2	2	0

For the Botox-enhanced SGB procedure, we injected 1 mL of 0.5% bupivacaine and 50 units of BOTOX® (onabotulinumtoxinA) into and around the sympathetic ganglion at the level of the right C6 anterior tubercle. We monitored the patient in the postop area for 30 minutes. During this observation, she developed signs of ipsilateral Horner's syndrome despite only 1 mL of local anesthetic injection around the stellate ganglion.

**Figure 1:**
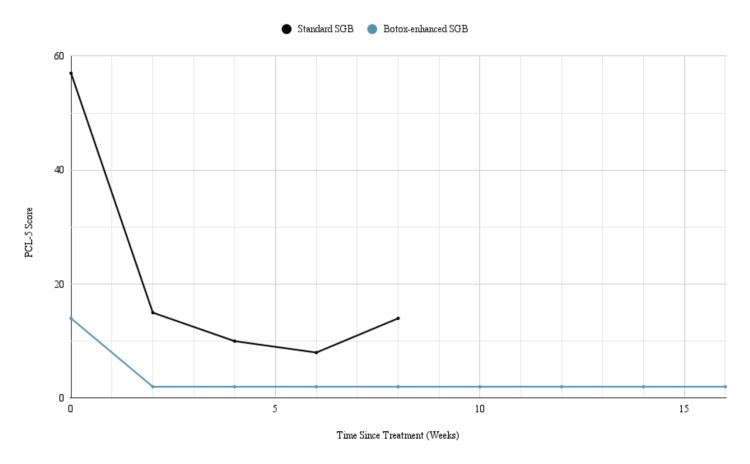
Monitoring PTSD symptoms over time To monitor the duration of post-traumatic stress disorder (PTSD) symptom relief, the patient completed the PTSD Checklist Version 5 (PCL-5) at regular intervals. The black line represents PCL-5 scores following the stellate ganglion block (SGB) treatments with 0.5% bupivacaine. The blue line represents PCL-5 scores following the subsequent treatments of SGB enhanced with 50 units of botulinum toxin (Botox). At the time of this publication, the patient continued to report significant symptom reduction with a sustained PCL-5 score of two with a local anesthetic alone.

Two weeks after the bilateral Botox-enhanced SGB, the patient reported significant improvement in symptoms of PTSD, sweating, insomnia, and anxiety. Her PCL-5 score was down to 2. While noting satisfactory symptom relief overall, she also reported a side effect of mild dysphagia. She rated the discomfort as a 3/10 on a standard pain scale and needed to drink fluids with food to aid swallowing. The dysphagia resolved after six weeks without additional intervention, and the patient did not experience any other adverse effects. At the six-month follow-up, the patient's PTSD and trauma-induced anxiety symptoms remained significantly reduced following the Botox-enhanced SGB treatments, indicating longer-lasting relief than the standard SGB (Figure 1).

## Discussion

The development of PTSD is typically related to a single traumatic event such as a car collision, a natural disaster, or a sexual assault. Researchers have suggested the current PTSD diagnosis does not fully capture the severe psychological harm that occurs when people experience repeated or prolonged traumatic exposure. In 2019, the World Health Organization’s eleventh revision of the International Disease Classification (ICD-11) identified complex PTSD (CPTSD) as a separate condition related to repeated or chronic trauma. The DSM-5 currently does not recognize additional diagnoses other than PTSD. The ICD-11 diagnosis characterizes CPTSD according to symptoms of affect dysregulation, negative self-concept, and disturbed relationships [13]. CPTSD symptoms can be similar but more enduring and extreme than those of PTSD. Some mental health professionals are beginning to distinguish between the two conditions and echoing the urgent demands for more research on treating this condition, despite the lack of guidance from the DSM-5 [14].

In this case, the patient reported a complex history of chronic trauma that continued for months and years, prompting her mental health professional to classify her symptoms as CPTSD. Because this ICD-11 designation is new, there are no clinical trials evaluating interventions for the treatment of individuals with complex presentations of PTSD. Several papers have discussed a multi-modular approach to the treatment of CPTSD, suggesting that these individuals may not benefit to the same degree from evidence-based treatments or may have higher rates of dropout from therapy [13]. Ultimately, current discussions support patient-centered care, emphasizing the importance of patient choice in identifying which problems to target, interventions to select, and outcomes to monitor [15]. These considerations guided the decision-making and treatment offerings throughout this case.

While this case reports promising results for Botox-enhanced SGB interventions, Botox itself can also induce some adverse effects. Commonly reported adverse effects of Botox injections include swelling or pain at the injection site, itching, and muscle weakness. In addition, recent studies have revealed systemic adverse effects of Botox, including anaphylaxis, dysphagia, and respiratory insufficiency, though most commonly observed in patients who receive high doses or have poor systemic conditions [7]. Clinical and laboratory data suggest that dysphagia following Botox therapy results from the spread of the toxin to adjacent pharyngeal muscles from the injection site [16]. One retrospective study assessed the occurrence of post-Botox injection dysphagia and found Botox to be equally safe and beneficial in patients with cervical dystonia, regardless of baseline risk of dysphagia [17]. Similarly, in this case, and in subsequent Botox-enhanced SGB treatments we performed, we found that transient dysphagia for several weeks is a commonly reported side effect. Most patients reported this as mildly uncomfortable and stated that drinking fluids with food aided in relieving this issue.

Further investigations with larger sample sizes, control groups, and more rigorous methodologies are needed to establish the efficacy and safety of Botox-enhanced SGB treatments. Additional research on the effects of psychotropic medications or continued psychotherapy following SGB treatment may provide a better understanding of each modality’s impact on patient outcomes. Despite the encouraging results from this case report, no other publications have described the injection of Botox into the stellate ganglion for PTSD or CPTSD treatment. The accumulation of more cases and longer-term follow-up are needed to confirm these findings and quantify the effect over time.

## Conclusions

While the standard SGB procedure has a strong safety and efficacy profile, a subset of PTSD patients, such as individuals with chronic complex presentations of PTSD, may not achieve sufficient or sustained symptom relief. This case study extends this work to suggest enhanced effectiveness in PTSD treatment by using Botox to supplement a traditionally approached sympathetic blockade of the stellate ganglion. The success, in this case, was measured by a sustained reduction in PCL-5 scores to date. These results warrant further investigation into the safety and clinical efficacy of Botox-enhanced SGB for PTSD compared to other SGB approaches. In this case, the patient expressed significant and profound results from SGB procedures for her PTSD when traditional psychiatric treatments had failed. We present Botox-enhanced SGB as a treatment for PTSD to augment and sustain the positive outcomes of a standard stellate ganglion blockade.
